# The Interaction of *Ocimum basilicum*, *Perilla frutescens* and *Mentha spicata* Essential Oils With Norfloxacin Against Antibiotic‐Resistant *Salmonella* Spp. That Cause Disease in Chickens

**DOI:** 10.1002/vms3.70316

**Published:** 2025-03-21

**Authors:** Nguyen Van Vui, Nguyen Thuy Linh, Nguyen Thi Kim Quyen, Kim Nang, Le Thi Tuyet Trinh

**Affiliations:** ^1^ Animal Science and Veterinary Medicine Department, Agriculture and Aquaculture Faculty Tra Vinh University Tra Vinh City Vietnam

**Keywords:** antibacterial, antibiotic‐resistance, essential oils, *Salmonella* spp, synergistic

## Abstract

**Background:**

The misuse of antibiotics in livestock farming has led to the emergence of antibiotic‐resistant bacteria, which pose a serious threat to global animal and human health. Essential oils extracted from the leaves of *Ocimum basilicum*, *Perilla frutescens* and *Mentha spicata* contain bioactive compounds with bactericidal properties.

**Objectives:**

This study aimed to evaluate the bactericidal activity of *Ocimum basilicum, Perilla frutescens* and *Mentha spicata* essential oils and their combination with the antibiotic norfloxacin, against antibiotic‐resistant *Salmonella* spp. isolated from diseased chickens.

**Methods:**

The antibiotic susceptibility testing of the isolated bacteria was conducted using the disc diffusion method. The bactericidal efficacy of essential oils and antibiotics was assessed by determining the minimum bactericidal concentration. The interaction between essential oils and antibiotics was analysed using the fractional bactericidal concentration index through the microdilution chequerboardtechnique.

**Results:**

*Salmonella typhimurium* and *Salmonella enteritidis* were recovered from the organs of infected chickens. Isolated *Salmonella* spp. displayed significant resistance to amoxicillin, ampicillin, streptomycin and cefuroxime. The essential oils of *Ocimum basilicum* and *Mentha spicata* demonstrated similar minimum bactericidal concentration (MBC) values of 8,000 µg/mL, while the essential oil of *Perilla frutescens* showed a higher MBC value of 10,000 µg/mL. Analysis of the interaction between these essential oils and norfloxacin indicated that both *Ocimum basilicum* and *Mentha spicata* exhibited a synergistic effect, whereas *Perilla frutescens* exhibited an additive effect when combined with norfloxacin in eradicating *Salmonella* spp.

**Conclusion:**

The study concluded that combining essential oils from three herbs with the antibiotic norfloxacin was highly effective in fighting antibiotic‐resistant *Salmonella* spp. isolated from infected chickens.

## Graphical abstract

This study demonstrates that *Ocimum basilicum*, *Perilla frutescens* and *Mentha spicata* essential oils, when combined with norfloxacin, effectively combat antibiotic‐resistant Salmonella spp. from infected chickens. Synergistic effects were observed with *Ocimum basilicum* and *Mentha spicata*, highlighting a potential solution to antibiotic resistance in livestock farming.

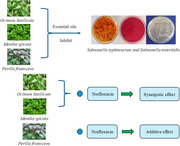

## 1 Introduction

Currently, poultry farming in Vietnam is gradually recovering and developing following avian influenza outbreaks. The increase in the number of poultry has increased the risk of disease outbreaks. In particular, the frequent and continuous occurrence of diarrhoea has caused significant losses, leading to poultry deaths, delayed market readiness, and heavy economic impacts on small‐scale farmers, large‐scale poultry operations and poultry breeding centres. There are many causes of diarrhoea in poultry, with *Salmonella* spp. being one of the primary pathogens. This bacterium is always present in the digestive system, faeces, feed, drinking water and bedding materials of chickens (Lutful Kabir 2010). When the immunity of chickens is compromised, the bacteria invade, proliferate and cause disease. Therefore, farmers have increasingly relied on daily antibiotics in feed and drinking water to prevent diseases. They have also overused antibiotics in treatments, which has contributed to antibiotic resistance in *Salmonella* spp. Numerous studies have shown that *Salmonella* spp. isolated from diseased poultry exhibit high antibiotic resistance (Castro‐Vargas et al. 2020; Tan et al. 2022), with some strains being multidrug‐resistant (Punchihewage‐Don et al. 2023). *Salmonella* spp. has also become resistant to several broad‐spectrum antibiotics, such as fluoroquinolones and cephalosporins. Furthermore, they can transfer antibiotic resistance from animal to human bacteria (Nair et al. 2018). *Salmonella* spp. is a major cause of diarrhoea in livestock and is among the most common causes of food poisoning worldwide. Given their widespread distribution, antibiotic‐resistant *Salmonella* spp. present a significant challenge for disease prevention, treatment and control, directly impacting public health owing to antibiotic residues in livestock products. This, in turn, leads to economic losses and health impacts and becomes a burden on humanity.

To control the harmful effects of pathogenic bacteria in poultry and antibiotic resistance in farming, many scientific studies have explored the use of essential oils extracted from herbs as an alternative to synthetic antibiotics (Galgano et al. 2022; Aouadhi et al. 2024). In Vietnam, aromatic herbs such as *Ocimum basilicum*, *Perilla frutescens* and *Mentha spicata* are widely used as food ingredients. In addition, these herbs are also utilised for extracting essential oils for medicinal purposes. All the essential oils from the three herbs contain useful bioactive compounds. The essential oil of *Ocimum basilicum* contains estragole (52.2%), linalool (16%) and 1,8‐cineole (7.4%) (Pandey et al. 2014); the essential oil of *Perilla frutescens* contains perillaldehyde (77.49%), limonene (5.61%) and β‐caryophyllene (4.89%) (Ahmed and Al‐Zubaidy 2020); the essential oil of *Mentha spicata* contains carvone (40.8%), limonene (20.8%) and 1,8‐cineole (17.0%) (Mahboubi 2021). Several studies have shown that these bioactive compounds possess antibacterial properties (Kamatou et al. 2008; Sharmaa et al. 2020; Angane et al. 2023). The bioactive compounds in essential oils can compromise the cell wall and cytoplasmic membrane, leading to lysis, leakage of intracellular contents, disruption of metabolic processes and ultimately cell death (Nazzaro et al. 2013). Therefore, these essential oils have great potential for killing *Salmonella* spp., which cause diarrhoea in chickens. Moreover, several studies have demonstrated that combining herbal essential oils with antibiotics can produce synergistic effects. The essential oils disrupt the bacterial cell membrane, facilitating the antibiotics' passage through the membrane and enabling them to reach effective concentrations more rapidly (Moussaoui and Alaoui 2016; Boonyanugomol et al. 2017; Romo‐Castillo et al. 2023; Drioiche et al. 2024). Consequently, combining these essential oils with antibiotics may counteract the antibiotic resistance of *Salmonella* spp. and reduce the dosage of antibiotics used in poultry farming. This study focused on assessing the bactericidal effects of essential oils from *Ocimum basilicum*, *Perilla frutescens* and *Mentha spicata*, both individually and in combination with an antibiotic, against antibiotic‐resistant *Salmonella* spp. isolated from infected chickens. This approach also addresses the urgent need to identify new development pathways that contribute to reducing farming costs, eliminating antibiotic residues and enhancing the value of livestock products.

## 2 Materials and Methods

### 2.1 Extraction and Analysis of Volatile Compounds in Essential Oils

*Ocimum basilicum, Perilla frutescens* and *Mentha spicata* were collected from an herb garden in Tra Vinh Province, Vietnam, in March 2023. These herbs were cultivated without pesticides or growth stimulants. Before use, all herbs were identified based on the morphology of the stem, branches and leaves (phenotype) by the Department of Crop Science, Tra Vinh University, Vietnam. Essential oils were extracted using steam distillation. After harvesting, the herbal leaves were dried at 40°C. Then, 150 g of dried leaves and 500 mL of water were placed into a 1000 mL round‐bottom flask of the Clevenger essential oil distillation system. The mixture was then heated to 100°C for 5 h. The essential oils were then separated from water using Na₂SO₄ and stored at 0–4°C until use.

The composition and relative content of volatile compounds in the essential oils were analysed using gas chromatography‐mass spectrometry (GC–MS). GC–MS analysis was carried out for each sample individually, utilising an Agilent Technology 6890N system equipped with an HP5–MS capillary column (30 m × 0.25 mm i.d. × 0.25 µm) and an Agilent Technologies MS 5973 Inert detector. The analysis began with an initial temperature of 50°C, held for 2 min, followed by a temperature increase of 5°C/min to 150°C, 10°C/min to 200°C and 20°C/min to 300°C, with a 5 min hold at the final temperature. Helium (1 mL/min) was used as the carrier gas and both the injector and detector were set to 250°C. The split ratio was 30:1. Prior to the injection of 0.2 µL, 10 µL of essential oils was diluted in 1000 µL of hexane. The essential oils composition was determined by calculating the GC peak areas. The mass spectra were acquired within the range of 40–400 m/z, with an ionisation energy of 70 eV. The mass spectra and linear retention indices (LRI) of the major components were compared with those from (NIST; Wiley) and cross‐checked against published mass spectra data (Adams 2007).

### 2.2 Culturing and Isolating *Salmonella* Spp

*Salmonella* spp. was isolated from 26 clinical samples (viscera and cloacal fluid). Samples were collected from diseased chickens at poultry farms in various districts of Tra Vinh Province, Vietnam. Upon dissection, the sampled chickens exhibited symptoms and lesions typical of *Salmonella* spp. infection, such as lethargy, emaciation, white diarrhoea, faeces adhering to the cloaca and white necrotic spots on the liver upon dissection. After collection, clinical samples were enriched in buffered peptone water (BPW) at 37°C for 24 h. The enriched bacterial solution was then selectively enriched in rappaport vassiliadis soya broth (RVS) and incubated at 37°C for 24 h. Subsequently, the bacterial solution was cultured on two media: Xylose‐Lysine Deoxycholate Agar (XLD) and MacConkey Agar (MC), to select colonies with the characteristic features of *Salmonella* spp.

### 2.3 Identifying Bacteria After Isolation

The bacteria, after isolation, were biochemically identified using the API 20E kit (Biomerieux, France) and the positive samples were subjected to gene sequencing using PCR.

### 2.4 Antibiotic Susceptibility Testing of Isolated *Salmonella* Spp

Isolated *Salmonella* spp. were tested for antibiotic susceptibility using the disc diffusion method described by (Bauer et al. 1966). Twelve antibiotics were used, including Amoxicillin 10µg (Ax), Tobramycin 10µg (Tb), Norfloxacin 10µg (Nr), Ceftazidime 30µg (Cz), Tetracycline 24µg (Te), Ampicillin 10µg (Am), Streptomycin 10µg (Sm), Kanamycin 30µg (Kn), Doxycycline 30µg (Dx), Colistin 10µg (Co), Gentamicin 10µg (Ge) and Cefuroxime 30µg (Cu). The results of antibiotic susceptibility tests were interpreted according to the Clinical and Laboratory Standards Institute for antimicrobial susceptibility testing (CLSI 2024). The experiment was repeated three times.

### 2.5 The Minimum Bactericidal Concentration (MBC) of Essential Oils (*Ocimum basilicum*, *Perilla frutescens* and *Mentha spicata*) and Antibiotic Against Isolated *Salmonella* Spp

MBC was determined using a broth microdilution susceptibility assay, as recommended by the Clinical and Laboratory Standards Institute for dilution antimicrobial susceptibility tests (CLSI 2024). The bacterial suspension was adjusted to a turbidity of 0.5 McFarland, corresponding to a concentration of 10^6^–10^8^ CFU/mL. Essential oils were diluted to concentrations ranging from 1 to 15 mg/mL, and the antibiotic norfloxacin was diluted to concentrations ranging from 13.25 to 4,000 µg/mL. The concentrations of essential oils and antibiotics used were based on preliminary experiment results to determine the concentration range capable of inhibiting bacteria. These concentrations of essential oils and antibiotics were added to the test tubes along with the bacterial suspension and incubated at 37°C for 24 h. After incubation, the solution from each test tube, corresponding to the concentrations of essential oils and antibiotics, was spread onto XLD agar and incubated at 37°C for another 24 h. The MBC of the essential oils and antibiotics against *Salmonella* spp. was determined as the lowest concentration in the test tube where no bacterial colonies grew on XLD medium. Each experiment was repeated thrice.

### 2.6 The Minimum Bactericidal Concentration (MBC) of Essential Oils (*Ocimum basilicum*, *Perilla frutescens* and *Mentha spicata)* and Antibiotic Used in Combination Against Isolated *Salmonella* Spp

The interaction between essential oils and antibiotics was evaluated using the fractional bactericidal concentration (FBC) index, following the microdilution chequerboard method described by (Moody 2003). At the minimum bactericidal concentration, essential oils and antibiotics were diluted in broth using a serial dilution method to 1/64 of the original concentrations. The MBC was determined for each combination of essential oil and antibiotic concentration.

FBC indices were calculated by summing FBC^A^ and FBC^B^, where FBC^A^ and FBC^B^ represent the minimum concentrations required to kill the bacteria for drugs A and B, respectively: FBC^A^ = MBC^A^ (in combination) / MBC^A^ (alone) and FBC^B^ = MBC^B^ (in combination) / MBC^B^ (alone). The mean FBC index was then determined using the formula: FBC index = FBC^A^ + FBC^B^, with the following classifications: synergistic (≤ 0.5), additive (> 0.5, but < 1), indifferent (≥ 1, but < 4), or antagonistic (≥ 4.0). The experiment was conducted three times with consistent results.

### 2.7 Statistical Analysis

Statistical analysis was performed using SPSS software version 22. The differences in MBC values between essential oils and antibiotics were compared using ANOVA followed by the Tukey test. A P‐value of less than 0.05 was considered statistically significant. Data were presented as mean ± SD.

## 3 Results

### 3.1 Yield and Composition of Essential Oil

After extraction by steam distillation, 1.1 mL of *Ocimum basilicum* essential oil with a yield of 0.74%, 0.55 mL of *Perilla frutescens* essential oil with a yield of 0.37% and 0.85 mL of *Mentha spicata* essential oil with a yield of 0.57% were obtained from 150 g of dried leaves of each herb. Table 1 presents the chemical composition of the essential oils from *Ocimum basilicum*, *Perilla frutescens* and *Mentha spicata which* was detected using the GC–MS method. In total, 30 compounds were identified in the essential oil of *Ocimum basilicum*, 23 in *Perilla frutescens* and 20 in *Mentha spicata*, collectively representing over 98% of the total compounds in these essential oils. The summary of the most abundant compounds in each essential oil is described in Table 2. The predominant components of *Ocimum basilicum* were estragole (52.65%), methyl eugenol (12.72%), 1,8‐cineole (6.72%), β‐linalool (5.34%), tau‐cadinol (3.74%) and trans‐α‐bergamotene (3.49%). In *Perilla frutescens*, the main constituents were perilla aldehyde (65.23%), β‐caryophyllene (8.32%), (3Z,6E)‐α‐farnesene (7.77%), D‐limonene (6.54%), estragole (1.17%), α‐linalool (1.30%) and perillyl alcohol (1.33%). The essential oil of *Mentha spicata* primarily contained carvone (48.90%), D‐limonene (17.60%), 1,8‐cineole (15.40%), β‐bourbonene (7.56%) and cis‐dihydrocarveol (1.24%).

**TABLE 1:** The composition of *Ocimum basilicum*, *Perilla frutescens* and *Mentha spicata* essential oils was determined using the GC–MS method.

		Relative percentage (%)		
Components	Retention time (min)	*Ocimum basilicum*	*Perilla frutescens*	*Mentha spicata*
3‐Octenol	10.080		0.6	
3‐Octanol	10.917		0.34	
𝛽‐Myrcene	11.960			0.42
D‐Limonene	12.635	0.42	6.54	17.6
Eucalyptol	12.726	0.65		
Menthone	13.340			1.15
1,8‐Cineol	13.490	6.72		15.4
cis‐β‐Ocimene	13.898	0.59		
Terpinen‐4‐ol	15.420			0.86
α‐Linalool	17.076		1.3	
β‐Linalool	17.101	5.34		0.3
Fenchol	17.689	0.86		
Camphor	19.346	0.96		
Borneol	20.461	0.54		
γ‐Terpinene	21.230			0.36
α‐Terpineol	21.699	0.48	0.45	0.53
Estragole	22.240	52.65	1.17	
Fenchyl acetate	22.972	0.68		
Dihydrocarveol	23.034			0.32
cis‐Dihydrocarveol	23.326			1.24
Dihydrocarvone	23.432			0.57
trans‐Carveol	23.540			0.4
Carvone	23.650			48.9
Dihydrocarvyl acetate	24.120			0.46
L‐Carveol	24.470			0.54
𝛽‐Bourbonene	24.870			7.56
Perilla aldehyde	25.177		65.23	
γ‐Amorphene	25.234			0.21
𝛼‐Amorphene	25.357			0.12
Bornyl acetate	25.467	0.26	0.45	
Perillyl alcohol	25.901		1.33	
Eugenol	27.852		0.5	
α‐Copaene	28.437		0.15	
β‐Elemene	28.949	1.21	0.11	
Methyl eugenol	29.324	12.72		
Isocaryophyllene	29.398		1.25	
β‐Caryophyllene	29.765	0.25	8.32	1.52
trans‐α‐Bergamotene	30.240	3.49		
α‐Guaiene	30.326	0.47		
Humulene	30.761	0.2	0.68	
cis‐Muurola‐4(15),5‐diene	31.029	0.56		
Germacrene D	31.521	0.64	0.55	
(3Z,6E)‐ α‐Farnesene	31.841		7.75	
Bicyclogermacrene	31.916	0.8	0.12	
δ‐Guaiene	32.134	0.45		
α‐Farnesene	32.142		0.75	
γ‐Cadinene	32.326	1.85		
δ‐Cadinene	32.522	0.62	0.26	
Nerolidol	33.333	0.15	0.25	
Spathulenol	33.685	0.23		
Caryophyllene oxide	33.804		0.46	0.3
Epicubenol	34.384	0.45		
tau‐Cadinol	34.830	3.74		
β‐Eudesmol	35.012	0.25		
α‐Cadinol	35.057	0.18		
Phytol	39.634		0.09	
Total		98.41	98.65	98.76

**TABLE 2:** The summarising the most abundant compounds in the essential oils of *Ocimum basilicum*, *Perilla frutescens* and *Mentha spicata*.

The predominant compounds found in the essential oils		
*Ocimum basilicum*	*Perilla frutescens*	*Mentha spicata*
Estragole (52.65%), Methyl eugenol (12.72%), 1,8‐Cineol (6.72%), β‐Linalool (5.34%), tau‐Cadinol (3.74%), trans‐α‐Bergamotene (3.49%), γ‐Cadinene (1.85%), β‐Elemene (1.21%), Camphor (0.96%), Fenchol (0.86%), Eucalyptol (0.65%), δ‐Cadinene (0.62%)	Perilla aldehyde (65.23%), β‐Caryophyllene (8.32%), (3Z,6E)‐ α‐Farnesene (7.75%), D‐Limonene (6.54%), Perillyl alcohol (1.33%), α‐Linalool (1.3%), Isocaryophyllene (1.25%), Estragole (1.17%), α‐Farnesene (0.75%), Humulene (0.68%), 3‐Octenol (0.6%), Eugenol (0.5%)	Carvone (48.9%), D‐Limonene (17.6%), 1,8‐Cineol (15.4%), 𝛽‐Bourbonene (7.56%), cis‐Dihydrocarveol (1.24%), β‐Caryophyllene (1.52%), Menthone (1.15%),, Terpinen‐4‐ol (0.86%), L‐Carveol (0.54%), α‐Terpineol (0.53%)

### 3.2 Bacterial Culture and Isolation

**FIGURE 1:**
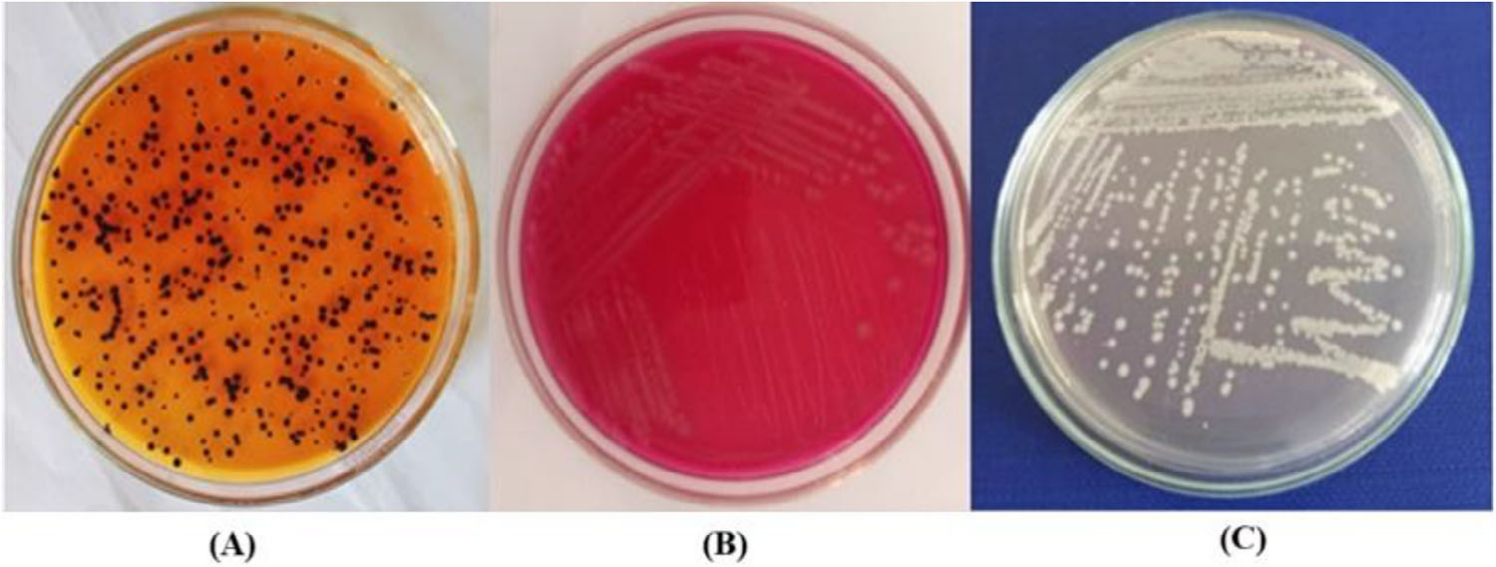
Suspected *Salmonella* spp. colonies on (A) Xylose‐Lysin deoxycholate agar, (B) MacConkey agar and (C) Tryptic soy agar.

The samples were initially enriched in BPW medium and then transferred to RVS selective enrichment medium. Both media showed turbidity, indicating a 100% positivity rate. From the RVS medium, bacteria were further cultured on XLD and MC media. The results were positive with colonies displaying a faint red halo with a black centre on the XLD medium and pink to red or beige colonies on the MC medium, both yielding a positive rate of 19.23%. The bacteria were then purified and subcultured on TSA medium to obtain biomass for biochemical identification and gene sequencing. The results of bacterial culture and isolation are presented in Table 3 and Figure 1.

**TABLE 3:** Results of culture and isolation *Salmonella* spp. from diseased chicken samples.

Bacterial culture media	Positive samples	Ratio (%)	Bacterial culture characteristics
BPW	26/26	100	Bacteria grow in a uniformly turbid medium
RVS	26/26	100	Bacteria grow in a uniformly turbid medium
XLD	05/26	19.23	Colonies have a light halo around a dark center
MC	05/26	19.23	Colonies are light pink or beige

Abbreviations: BPW: Buffered Peptone Water; RVS: Rappaport Vassiliadis Soya Broth; XLD: Xylose‐Lysin Deoxycholate Agar; MC: MacConkey Agar.

### 3.3 Bacterial Identification Using the API 20E Biochemical Kit

The bacterial identification results using the API 20E biochemical kit are shown in Table 4. The results indicate that out of five test samples, two tested positive for *Salmonella* spp., which were isolated from the internal organs of diseased chickens.

**TABLE 4:** Biochemical identification results of the isolated bacteria using the API 20E kit.

Type of samples	Identification code determined	Identified bacterial species
Viscera	6717556	*Salmonella enterica*
Viscera	6727552	*Salmonella enterica*
Cloacal fluid	7266542	*Aeromonas hydrophila*
Cloacal fluid	2576000	*Proteus mirabilis*
Cloacal fluid	2537000	*Proteus mirabilis*

### 3.4 Identification of Isolated Bacteria Using PCR

The results of bacterial identification by PCR are recorded in Table 5. The findings indicate that out of five test samples, two samples tested positive for *Salmonella enterica* subsp. *Enterica* (99.20%) and *Salmonella enterica* subsp. *Enterica* (99.37%). A comparison of the sequenced genes with the NCBI database showed 99.06% similarity and 100% coverage with *Salmonella enterica* subsp. *Enterica* serovar Typhimurium, 99.66% similarity and 100% coverage with *Salmonella enterica* subsp. *Enterica* serovar Enteritidis.

**TABLE 5:** Identification results of isolated bacteria using PCR technique.

Type of samples	Number of samples	Positive samples	Identified bacterial species
Viscera	2	02	*Salmonella enterica*
Cloacal fluid	3	00	—

### 3.5 Antibiotic Susceptibility Test Results for Isolated *Salmonella* Spp

**FIGURE 2:**
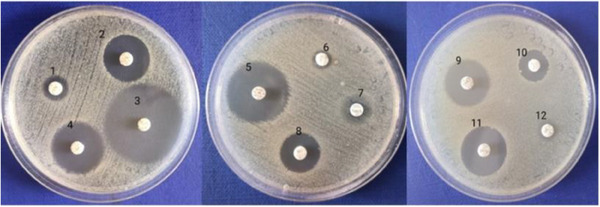
The image of antibiotic resistance in isolated *Salmonella* bacteria. (1) Amoxicillin, (2) Tobramycin, (3) Norfloxacin, (4) Ceftazidime, (5) Tetracycline, (6) Ampicillin, (7) Streptomycin, (8) Kanamycin, (9) Doxycycline, (10) Colistin, (11) Gentamycin and (12) Cefuroxime.

The results of the antibiotic susceptibility testing of the isolated *Salmonella* spp. are shown in Table 6. The antibiotic susceptibility profiles of the two isolated bacteria, *Salmonella typhimurium* and *Salmonella enteritidis*, were identical in terms of sensitivity and resistance to the tested antibiotics. The results show that among the 12 antibiotics tested, the isolated *Salmonella* spp. was sensitive to norfloxacin, tetracycline, ceftazidime, gentamycin, doxycycline and tobramycin. Among these, the bacteria showed the highest sensitivity to norfloxacin. Therefore, norfloxacin was selected in combination with essential oils to determine the bactericidal role of the interaction between the essential oils and antibiotics in the next experiment. However, the isolated *Salmonella* spp. exhibited high insensitivity to amoxicillin, ampicillin, streptomycin, cefuroxime and colistin; and complete resistance to amoxicillin, ampicillin, streptomycin and cefuroxime. Antibiotic susceptibility profiles of the isolated *Salmonella* spp. are depicted in Figure 2.

**TABLE 6:** Antibiotic susceptibility results of the isolated *Salmonella* spp.

		Zone of growth inhibition (mm)					
		*Salmonella typhimurium*			*Salmonella enteritidis*		
Antibiotics	Levels (µg)	S (≥21)	I (18‐20)	R (≤17)	S (≥21)	I (18‐20)	R (≤17)
Ampicillin (Am)	10			00			00
Amoxicillin (Ax)	10			00			00
Ceftazidime (Cz)	30	22 ± 0.58				20 ± 1.00	
Cefuroxime (Cu)	30			00			00
Colistin (Co)	10			8 ± 1.15			00
Gentamycin (Ge)	10		19 ± 1.53			20 ± 0.58	
Streptomycin (Sm)	10			00			00
Tetracycline (Te)	30	24 ± 1.00			22 ± 1.15		
Doxycycline (Dx)	30		18 ± 1.00		22 ± 0.58		
Tobramycin (Tb)	10		18 ± 1.53			20 ± 1.15	
Kanamycin (Kn)	30			12 ± 0.58			13 ± 1.00
Norfloxacin (Nr)	10	47 ± 1.00			50 ± 1.15		

Abbreviations: S: sensitive; I: intermediate; R: resistant. The values represent mean ± SD.

### 3.6 Antibacterial Activity of Essential Oils (*Ocimum basilicum*, *Perilla frutescens* and *Mentha spicata*) and Norfloxacin Used Alone and in Combination Against Isolated *Salmonella* Spp

After testing the antibiotic resistance of the isolated bacteria, norfloxacin was found to be the most effective antibiotic against the bacteria and was therefore used for the subsequent experiments. Table 7 presents the bactericidal effects of the essential oils and antibiotics, as well as the interaction between the essential oils and norfloxacin when used in combination against isolated *Salmonella* spp. The results indicated that the effects of the essential oils and antibiotics on the two strains, *Salmonella typhimurium* and *Salmonella enteritidis*, were similar. The essential oils of *Ocimum basilicum* and *Mentha spicata* exhibited similar MBC values of 8,000 µg/mL, whereas the essential oil of *Perilla frutescens* had a higher MBC value of 10,000 µg/mL. This suggests that the bactericidal efficacy of *Perilla frutescens* against *Salmonella* spp. was lower than that of *Ocimum basilicum* and *Mentha spicata*. Norfloxacin demonstrated very high bactericidal efficacy with an MBC value of 62.5 µg/mL. Although the MBC values of the essential oils were 128–160 times higher than that of norfloxacin, all essential oils exhibited antibacterial activity against the isolated *Salmonella* spp. The results of the interaction between the essential oils and norfloxacin revealed that both *Ocimum basilicum* and *Mentha spicata* essential oils had a synergistic effect when combined with norfloxacin, while *Perilla frutescens* showed an additive effect in killing *Salmonella* spp. in combination with norfloxacin. The minimum bactericidal concentration of norfloxacin against the isolated *Salmonella* spp. decreased by 32 times when combined with *Ocimum basilicum* essential oil, by 16 times when combined with *Mentha spicata* essential oil and by 8 times when combined with *Perilla frutescens* essential oil. Additionally, the MBC values of the essential oils were also significantly reduced when combined with norfloxacin at 3,000 µg/mL (*Ocimum basilicum*), 2,000 µg/mL (*Mentha spicata*) and 8,000 µg/mL (*Perilla frutescens*).

**TABLE 7:** The minimum bactericidal concentration (MBC) and fractional bactericidal concentration index (FBC) of essential oils (*Ocimum basilicum*, *Perilla frutescens* and *Mentha spicata)* and Norfloxacin used alone and in combination against isolated *Salmonella* spp.

Compounds		*Salmonella typhimurium*			*Salmonella enteritidis*		
		MBC (µg/ml)	FBC (index)	Type of interaction	MBC (µg/ml)	FBC (index)	Type of interaction
Alone	OB	8,000 ± 100^b^			8,000 ± 150^b^		
	MS	8,000 ± 200^b^			8,000 ± 100^b^		
	PF	10,000 ± 500^c^			10,000 ± 100^c^		
	NR	62.50 ± 2.50^a^			62.50 ± 1.00^a^		
Combination	OB + NR	3,000/1.95	0.40	Synergism	3,000/1.95	0.40	Synergistic
	MS + NR	2,000/3.90	0.31	Synergism	2,000/3.90	0.31	Synergistic
	PF + NR	8,000/7.81	0.92	Additive	8,000/7.81	0.92	Additive

Abbreviations: *OB: Ocimum basilicum*, PF: *Perilla frutescens*, MS: *Mentha spicata*, Nr: Norfloxacin. Superscript letters (a, b and c) within the same column indicate statistically significant differences (P < 0.05). The values represent mean ± SD.

## 4 Discussion

The research results showed that the internal organ specimens collected from the field tested positive for *Salmonella* spp., specifically with the two strains *Salmonella typhimurium* and *Salmonella enteritidis*. In contrast, the specimens collected from cloacal fluid tested negative for *Salmonella* spp. This can be explained by the fact that cloacal fluid samples may contain a variety of intestinal bacteria, which are highly competitive and grow rapidly, making it difficult to isolate *Salmonella* spp. from these samples compared to those collected from internal organs. This finding is consistent with that of Shen et al. (2023), who also isolated two strains of *Salmonella typhimurium* and *Salmonella enteritidis* from the internal organs of diseased chickens. The bacterial identification results using the API 20E biochemical kit and gene sequencing were consistent, demonstrating the high reliability of the API 20E biochemical kit, which is recommended for preliminary screening before using gene sequencing for bacterial identification. Antibiotic susceptibility test results showed that the two isolated *Salmonella* spp. strains were completely resistant to the antibiotics amoxicillin, ampicillin, streptomycin and cefuroxime. These antibiotics are commonly available in the market today in both veterinary and human medicine. These results are consistent with those of previous studies on antibiotic resistance of *Salmonella* spp. strains isolated from diseased chickens (Castro‐Vargas et al. 2020; Tan et al. 2022; Punchihewage‐Don et al. 2023). This poses a significant challenge that humanity must face and requires attention and research to develop new antimicrobial agents from various sources, including essential oils extracted from herbs, to combat the antibiotic resistance of microorganisms. Moreover, the antibiotic susceptibility test results for the two isolated *Salmonella* spp. strains were identical in terms of sensitivity and resistance to the tested antibiotics. This may be due to the fact that the specimens were collected from the same locality and the animals were under the same veterinary network. The frequent use of certain antibiotics in feed, water, or during disease prevention and treatment by veterinarians and livestock farmers over an extended period has led to the development of similar antibiotic resistance in some microorganisms, including *Salmonella* spp.

In the present study, essential oils from three herbs, *Ocimum basilicum*, *Perilla frutescens* and *Mentha spicata*, were used to evaluate their bactericidal activities against isolated *Salmonella* spp. The results showed that all three essential oils were capable of killing isolated *Salmonella* spp. The bactericidal activity of these three essential oils may be due to the chemical components present in the essential oils that possess bactericidal properties or the synergistic effect of bioactive compounds, which contribute to enhancing the bactericidal activity. Many previous studies have demonstrated that the bioactive compounds in these three essential oils have high bactericidal potential. The essential oils of *Ocimum basilicum* and *Perilla frutescens* contain estragole, linalool and thymol, which can inhibit *Shigella flexneri* (Ngome et al. 2018). In addition, 1,8‐cineole, an active compound found in the essential oils of *Ocimum basilicum* and *Mentha spicata*, exerts bactericidal activity against *Klebsiella pneumoniae* (Moo et al. 2021) and *Escherichia coli* (Wang et al. 2022). Moreover, carvone and D‐limonene, which are present in both *Perilla frutescens* and *Mentha spicata* essential oils, have been shown to kill common bacteria that affect humans and livestock (Jacob et al. 2017; Agougui et al. 2022). Eugenol, found in *Ocimum basilicum* essential oil, also exerts bactericidal activity against *S. aureus* and *E. coli* (Jeyakumar and Lawrenceb 2021; Bai et al. 2023).

The results indicated that the bactericidal effectiveness of the three essential oils varied, with *Ocimum basilicum* and *Mentha spicata* showing higher bactericidal activity than *Perilla frutescens*. The differences in bactericidal effectiveness among these essential oils can be explained by the diversity of bioactive compounds present in each oil, which leads to different mechanisms of action against bacteria. Additionally, the synergistic or inhibitory effects of bioactive compounds within essential oils also influence their bactericidal effectiveness. Previous studies have identified the synergistic interactions or inhibitory effects of bioactive compounds on the bactericidal activity of herbal essential oils (Kamatou et al. 2008; Sharmaa et al. 2020; Angane et al. 2023). The bioactive compounds in essential oils are hydrophobic, which disrupts the lipid components of bacterial cell membranes and mitochondria, increasing their permeability. This disruption leads to the breakdown of the cell wall and cytoplasmic membrane, causing lysis and leakage of intracellular compounds, adversely affecting metabolism and resulting in cell death (Nazzaro et al. 2013). Furthermore, components of essential oils affect bacterial intracellular proteins, impacting cell division (Domadia et al. 2007). Although gram‐negative bacteria have higher resistance to essential oils than gram‐positive bacteria because of the unique structure of their cell walls, which include an additional outer membrane linked to peptidoglycan by lipoproteins, this double membrane limits the effects of essential oils (Swamy et al. 2016). However, eugenol and isoeugenol in *Ocimum basilicum* and *Mentha spicata* demonstrate higher activity against gram‐negative bacteria than gram‐positive bacteria because they can alter the bacterial membrane, affect ion and ATP transport and modify fatty acid structures. Eugenol also acts against various bacterial enzymes, including ATPase, histidine carboxylase, amylase and proteases (Jeyakumar and Lawrence 2021). In contrast, *Perilla frutescens* contains perillaldehyde as its primary active compound, which is less effective in penetrating bacterial cells and damaging their internal components *Salmonella* spp. (Dorman and Deans 2000). Moreover, it has been explained that the bioactive compounds in *Ocimum basilicum* and *Mentha spicata* work synergistically, enhancing their overall antibacterial effects. For instance, these compounds can weaken the bacterial membrane, facilitating the entry of other compounds into the cell to disrupt metabolic processes. In *Perilla frutescens*, the bioactive compounds do not exhibit the same level of synergy, reducing their combined effectiveness. This explains why the MBC values of *Ocimum basilicum* and *Mentha spicata* were lower than that of *Perilla frutescens*.

The combination of essential oils with norfloxacin reduced the MBC values of both the essential oils and the antibiotic against the isolated *Salmonella* spp. The combination of *Ocimum basilicum* and *Mentha spicata* essential oils with the antibiotic resulted in a synergistic interaction, whereas the combination of *Perilla frutescens* essential oil with the antibiotic resulted in an additive interaction. This outcome is consistent with previously published studies on the synergistic interactions between essential oils and antibiotics (Moussaoui and Alaoui 2016; Boonyanugomol et al. 2017; Romo‐Castillo et al. 2023; Drioiche et al. 2024). The minimum bactericidal concentration of norfloxacin against *Salmonella* spp. was significantly reduced when combined with the essential oils. Experimental results and published research suggest that combining essential oils with antibiotics can reduce the amount of antibiotics needed and decrease bacterial resistance to antibiotics. In other words, the combination of essential oils and antibiotics increases the sensitivity of bacteria to antibiotics, thereby opening the potential for developing new therapies to combat multidrug‐resistant pathogens. The synergistic effect of *Ocimum basilicum* and *Mentha spicata* essential oils when combined with norfloxacin is due to their effective support in the process of killing *Salmonella* spp. Norfloxacin, a second‐generation antibiotic in the quinolone group, penetrates bacterial cells and inhibits DNA gyrase, an enzyme essential for DNA replication in *Salmonella* spp. This penetration was more effective with the support of antibacterial components in *Ocimum basilicum* and *Mentha spicata* essential oils. These components disrupt the bacterial cell membrane, allowing norfloxacin to be transported more easily across the membrane and quickly reach an effective concentration. Simultaneously, the increased inhibition of *Salmonella* spp. cells by norfloxacin reduced the amount of *Ocimum basilicum* and *Mentha spicata* essential oils required to achieve bactericidal activity. On the other hand, the combination of *Perilla frutescens* essential oil with norfloxacin showed additive interactions in the process of killing *Salmonella* spp., as they performed the same task without supporting each other in the bactericidal process. Perillaldehyde in *Perilla frutescens* essential oil can reach convoluted and deeper parts of the cell, depleting intracellular ATP, but it does not disrupt the outer membrane of *Salmonella* spp. cells (Dorman and Deans 2000). Thus, it does not support norfloxacin permeability through the cell membrane and vice versa. They simply inhibit and kill *Salmonella* spp.; therefore, the concentration needed in combination is lower than when used alone, resulting in only an additive interaction.

## 5 Conclusions

The study concluded that essential oils from *Ocimum basilicum* (8 mg/mL), *Mentha spicata* (8 mg/mL) and *Perilla frutescens* (10 mg/mL) demonstrated the ability to kill antibiotic‐resistant *Salmonella spp*. isolated from diseased chickens in the field under in vitro conditions. The combination of *Ocimum basilicum* and *Mentha spicata* essential oils with the antibiotic norfloxacin exhibited a synergistic effect in eliminating *Salmonella spp*., whereas *Perilla frutescens* essential oil showed an additive effect when combined with the antibiotic. Further in vivo studies are needed to confirm these findings under real poultry production conditions, paving the way for recommendations to incorporate these essential oils into poultry disease treatment regimens. This approach could offer benefits such as reduced dosages, minimised side effects and decreased antibiotic resistance in the future.

## Author Contributions

**Nguyen Van Vui**: conceptualisation, investigation, writing—original draft, methodology, writing—review and editing, formal analysis, data curation, supervision. **Nguyen Thuy Linh**: methodology, writing—review and editing, data curation. **Nguyen Thi Kim Quyen**: methodology, writing—review and editing, data curation. **Kim Nang**: methodology, formal analysis, data curation. **Le Thi Tuyet Trinh**: methodology, formal analysis, data curation.

## Ethics Statement

The authors confirm that the ethical policies of the journal, as noted on the journal's author guidelines page, have been adhered to and the appropriate ethical review committee approval has been received. The procedures conducted in this study were approved by the Care and Use of Laboratory Animals Committee of Tra Vinh University, Vietnam, under license number 273/2021/HD.HĐKH&DT‐ĐHTV.

## Conflicts of Interest

The authors declare no conflicts of interest.

### Peer Review

The peer review history for this article is available at https://www.webofscience.com/api/gateway/wos/peer‐review/10.1002/vms3.70316.

## Data Availability

The data that support the findings of this study are available from the corresponding author upon reasonable request.
